# Assessment of early treatment response to neoadjuvant chemotherapy in breast cancer using non-mono-exponential diffusion models: a feasibility study comparing the baseline and mid-treatment MRI examinations

**DOI:** 10.1007/s00330-016-4630-x

**Published:** 2016-10-31

**Authors:** Reem Bedair, Andrew N. Priest, Andrew J. Patterson, Mary A. McLean, Martin J. Graves, Roido Manavaki, Andrew B. Gill, Oshaani Abeyakoon, John R. Griffiths, Fiona J. Gilbert

**Affiliations:** 1 Department of Radiology, School of Clinical Medicine, University of Cambridge, Box 218, Cambridge Biomedical Campus, Hills Road, Cambridge, CB2 0QQ UK; 2 Department of Radiology, Addenbrookes Hospital, Cambridge University Hospitals NHS Foundation Trust, Hills Road, Cambridge, CB2 0QQ UK; 3 Cancer Research UK Cambridge Institute, University of Cambridge, Li Ka Shing Centre, Cambridge, CB2 0RE UK

**Keywords:** Breast carcinoma, Diffusion-weighted MRI, Neoadjuvant treatment, Quantitative evaluation, Tumour biomarkers

## Abstract

**Objectives:**

To assess the feasibility of the mono-exponential, bi-exponential and stretched-exponential models in evaluating response of breast tumours to neoadjuvant chemotherapy (NACT) at 3 T.

**Methods:**

Thirty-six female patients (median age 53, range 32–75 years) with invasive breast cancer undergoing NACT were enrolled for diffusion-weighted MRI (DW-MRI) prior to the start of treatment. For assessment of early response, changes in parameters were evaluated on mid-treatment MRI in 22 patients. DW-MRI was performed using eight *b* values (0, 30, 60, 90, 120, 300, 600, 900 s/mm^2^). Apparent diffusion coefficient (ADC), tissue diffusion coefficient (*D*_t_), vascular fraction (ƒ), distributed diffusion coefficient (DDC) and alpha (α) parameters were derived. Then *t* tests compared the baseline and changes in parameters between response groups. Repeatability was assessed at inter- and intraobserver levels.

**Results:**

All patients underwent baseline MRI whereas 22 lesions were available at mid-treatment. At pretreatment, mean diffusion coefficients demonstrated significant differences between groups (*p* < 0.05). At mid-treatment, percentage increase in ADC and DDC showed significant differences between responders (49 % and 43 %) and non-responders (21 % and 32 %) (*p* = 0.03, *p* = 0.04). Overall, stretched-exponential parameters showed excellent repeatability.

**Conclusion:**

DW-MRI is sensitive to baseline and early treatment changes in breast cancer using non-mono-exponential models, and the stretched-exponential model can potentially monitor such changes.

***Key points*:**

• *Baseline diffusion coefficients demonstrated significant differences between complete pathological responders and non-responders.*

• *Increase in ADC and DDC at mid-treatment can discriminate responders and non-responders.*

• *The ƒ fraction at mid-treatment* decreased *in responders whereas* increased *in non-responders.*

• *The mono- and stretched-exponential models showed excellent inter- and intrarater repeatability.*

• *Treatment effects can potentially be assessed by non-mono-exponential diffusion models.*

**Electronic supplementary material:**

The online version of this article (doi:10.1007/s00330-016-4630-x) contains supplementary material, which is available to authorized users.

## Introduction

Neoadjuvant chemotherapy (NACT) has increasingly been utilized in the treatment of breast cancer to enable breast-conserving surgery and improve resectability. Approximately 80 % of patients have been found to respond to NACT, but only 6–25 % of patients show complete pathological response (pCR) [1–3]. Therefore, functional imaging techniques have been investigated for the prediction of response early after initiating therapy.

Diffusion-weighted magnetic resonance imaging (DW-MRI) derives image contrast from the differences in the mobility of water protons between tissues. DW imaging in breast protocols generally involves the acquisition of images at two *b* values to quantitatively determine the mono-exponential relationship between signal attenuation and *b* value. The apparent diffusion coefficient (ADC) enables characterisation of lesions based on differences in water diffusivity which in turn reflect tissue cellularity and integrity of membranes [4]. The association with cellular density makes ADC ideal for monitoring cytotoxic responses [5].

However, it has been shown that the degree of signal attenuation in breast tissue becomes non-linear with increasing *b* value. Bi-exponential signal decay has been observed over a range of *b* values, where a small increase (0 − 200 s/mm^2^) results in a steep reduction in the measured signal intensity. This has been related to perfusion in the microcapillary circulation (expressed as pseudo-diffusion coefficient *D*_p_ and vascular fraction ƒ). The signal then attenuates more gradually over the range of higher *b* values (>200 s/m^2^) enabling the measurement of true tissue diffusivity (*D*_t_). This phenomenon, known as intravoxel incoherent motion (IVIM), enables the separation of molecular diffusion from perfusion, provided that a wide range of low and high *b* values are used [6].

Whilst the IVIM model has the advantage that its components can be associated with distinct physical phenomena [7, 8], estimates of perfusion have not been fully investigated in practice for the assessment of therapeutic response in breast cancer.

To overcome the assumptions associated with the bi-exponential model, Bennett et al. introduced the stretched-exponential approach, which models the continuous distribution of diffusion compartments attenuating at different rates (termed DDC, distributed diffusion coefficient). The plot of the signal intensity vs. *b* value becomes characteristically stretched, indicating deviation from the single exponential decay. This is denoted by the parameter α, which provides a new type of image contrast that relates to the degree of intravoxel water diffusion heterogeneity ranging from 0 to 1. A numerically high α index (i.e. α approaching 1) represents low intravoxel heterogeneity indicative of mono-exponential diffusion-weighted signal decay, whereas a numerically low α index (i.e. approaching 0) represents a high degree of diffusion heterogeneity exhibited as multi-exponential signal decay [9–11].

In general, these non-Gaussian diffusion models offer more parameters, which provide a better fit to the diffusion data. Preliminary studies suggest that multi-exponential models implemented in tumours of the brain [12, 13], head and neck [14, 15], abdomen [16] and prostate [17–19] can offer additional information on tissue heterogeneity, vascularity and cellularity beyond ADC.

The purpose of this work was to assess the feasibility of diffusion parameters obtained from the mono-exponential, bi-exponential and stretched-exponential diffusion models in evaluating response of breast tumours to NACT at 3 T.

## Materials and methods

### Patient population and study design

The local institutional review boards and ethics committees approved this prospective study and written informed consent was obtained from all patients.

Patients were eligible if they were at least 18 years of age, had pathologically confirmed invasive breast cancer and were undergoing NACT as a first line of treatment. Patients were ineligible if they had poor renal or liver functions, allergy to gadolinium-based contrast agents, metals implants or a pacemaker.

Assuming a moderate effect size between the diagnostic performance of diffusion parameters and response (effect size = 0.6), a sample size of 19 patients would be needed to yield a power of 80 % with 95 % confidence levels. Between February 2014 and September 2015, 40 female patients (median age 55, range 32 − 75 years) presented with a palpable lump and underwent core biopsies under ultrasound guidance by an experienced radiologist in the outpatient clinic prior to the start of treatment. According to our local protocol, patients received six cycles of NACT. The regimen consisted of docetaxel 100 mg/m^2^ once every 21 days for three cycles, followed by fluorouracil 500 mg/m^2^, epirubicin 100 mg/m^2^, with cyclophosphamide 500 mg/m^2^ (FEC) once every 21 days for three cycles if the tumour was negative for human epidermal growth factor 2 (HER2−) on biopsy. Two patients, however, were started on weekly taxols for 12 weeks at a lower dose because of their age, as studies show that weekly doses may offer the same benefit with fewer side effects [20, 21]. They subsequently received the FEC part of their treatment as described. This combination was reversed with the HER2+ cancers. In addition, docetaxel was combined with the HER2-targeted agent trastuzumab (Herceptin®; Genentech Inc., CA) in these patients for the last three cycles. This therapeutic combination is based on the recommendation of the international consensus conference for neoadjuvant systemic therapy in primary breast cancer [1]. Patient and tumour characteristics are summarized in Table 1.

**Table 1:** Tumour characteristics and patient outcome

Characteristic	Responder	Non-responder
Age (median age, range)	51 (36−66)	53 (42−68)
Tumour size		
<2 cm	0	1
2–5 cm	11	10
>5 cm	3	11
Morphology		
Mass	13	12
Non-mass like	1	10
Tumour histology		
Invasive ductal carcinoma	12	18
Papillary carcinoma	1	1
Mucinous	0	1
Medullary	1	0
Mixed carcinoma^a^	0	2
Histological grade		
II	3	14
III	11	8
Oestrogen receptor (ER) status^b^		
Positive (+)	4	20
Negative (−)	10	2
HER-2/neu receptor status^c^		
Positive (+)	2	11
Negative (−)	12	11

Unless otherwise indicated, data are number of patients

^a^Histology showed invasive carcinoma of mixed types; one of which was mucinous and ductal type, and the other showing lobular growth pattern with tubule formation classified as mixed lobular and ductal carcinoma

^b^Tumours were classified as oestrogen receptor positive (and progesterone receptor positive) if more than 10 % of the cells were stained positively

^c^Tumours were classified as human epidermal growth factor 2 (HER2) positive when they scored 3+ at immunohistochemistry or when gene amplification was observed with fluorescence in situ hybridization (FISH)

### MRI technique

All MR examinations were performed on a 3.0-T system (MR750, GE Healthcare) with a dedicated eight-channel breast array coil. The MRI protocol included T_1_- and T_2_-weighted axial images, DWI and dynamic contrast-enhanced (DCE) series (Table 2). DW-MRI was performed utilizing a single-shot spin-echo echo planar imaging sequence at eight *b* values (0, 30, 60, 90, 120, 300, 600 and 900 s/mm^2^).

**Table 2:** MRI sequence parameters

Parameters	T_2_-weighted	Diffusion-weighted imaging	Contrast-enhanced T_1_-weighted
Sequence	SE	2D SS-EPI	3D SPGR
FOV (mm^2^)	350 × 350	350 × 350	350 × 350
Image matrix	384 × 256	128 × 128	512 × 512
Section thickness (mm)	4.0	4.0	2.8 (interpolated to 1.4)
*b* values (s/mm^2^)	–	0, 30, 60, 90, 120, 300, 600, 900	–
Pixel size (mm^2^)	0.9 × 1.3	2.7 × 2.7	0.6 × 0.6
Fat suppression	No	Spatial-spectral water excitation with water spectral presaturation	Spatial-spectral water excitation
Parallel acquisition (ASSET factor)	No	2	2.5 (phase direction)
TR (ms)	4.6	5.0	7.1
TE (ms)	76.2	77.9	3.7
RF excitation (degrees)	111	90	12
No. of averages	1	5	0.5
Bandwidth (kHz)	62.5	250	125
No. of slices	38	40	112
Acquisition time	47 s	9 min	8 min 7 s

*SE* spin-echo, *2D SS-EPI* 2-dimensional single-shot echo planar imaging, *3D SPGR* 3-dimensional spoiled gradient-recalled echo, *FOV* field of view, *ASSET* array coil spatial sensitivity encoding technique, *TR* repetition time, *TE* echo time, *RF* radiofrequency

Subsequently, DCE-MRI data was acquired using a three-dimensional segmented *k*-space spoiled gradient-echo technique. Five acquisitions were obtained before contrast agent injection and then once every 10 s after bolus injection of 0.1 mmol/kg of gadopentetate dimeglumine (Magnevist, Bayer Schering, Berlin) for 8 min 7 s. The total examination time was approximately 25 min.

Patients underwent MR imaging before the start of chemotherapy, after completion of three cycles and at the end of therapy. For the purposes of assessing early response, data from the baseline and midway scans only were analysed. The baseline MRI was performed approximately 10 days after histological confirmation of malignancy (range 7–11 days). The median time interval between pretreatment MRI and the start of therapy was 1 day (range 0–2).

The median interval between the third cycle of chemotherapy and the second MRI was 21 days (range 15–22).

After completion of NACT, all patients received breast and axillary surgery, radiotherapy and endocrine therapy as appropriate.

### Image analysis

Tumours were identified on the post-contrast T_1_-weighted images by two breast radiologists in consensus (R.B, F.J.G, Cambridge, UK) with 4 and 20 years of experience in breast MRI. Lesion site, size, morphology (mass vs. non-mass-like lesion), enhancement pattern (heterogeneous vs. homogeneous enhancement) and kinetic features (curve type; type I (progressive), type II (plateau) or type III (washout)) were recorded. For both mass and non-mass-like lesion demarcation, the early subtracted DCE images were used as reference where freehand regions of interest (ROIs) were manually drawn on the imaging slice with the largest tumour dimension on the *b* = 900 s/mm^2^ image by one radiologist (R.B). Care was taken to avoid tumour borders and areas of necrosis. Intraobserver variability was evaluated by redrawing the ROIs 3 weeks after the initial measurements. A third radiologist (O.A) with 3 years’ experience in breast MRI manually reoutlined the tumours to assess the inter-rater variability of measurements.

All readers were blinded to the pathological findings and therapeutic responses.

Diffusion analyses were performed using in-house software developed in MATLAB (The Mathworks, Natick, MA). To enable comparison with previous DWI breast studies, the ADC was calculated from two *b* values (0, 900 s/mm^2^). All *b* values were used for the stretched-exponential and IVIM models. For the non-Gaussian models, data was fitted using a non-linear least-squares approach. ROIs were analysed on a voxel-wise basis and parameters were expressed as means over the volumes measured. Parametric maps of diffusion coefficients were generated. Details of the quantitative diffusion models are provided in the supplementary material. Percentage change in parameters was calculated as: (Parameter_mid_ − Parameter_pre_)/Parameter_pre_ × 100, where Parameter_pre_ and Parameter_mid_ are the baseline and mid-treatment measurements respectively.

### Histological analysis and response assessment

Histopathological assessment was performed after surgical excision following the last cycle of chemotherapy. Tumour type, grade, hormone-receptor status and HER2 expression were obtained from reports of the core biopsies or surgical specimens. Although molecular testing is prognostic, it is expensive and not widely available. Therefore according to Onitilo et al. [22, 23], four immunohistochemical (IHC) categories have been identified on the basis of the hormonal-receptor status (oestrogen and progesterone receptor ER, PR) and HER2 overexpression. These groups are ER+PR+/HER2−, ER+PR+/HER+, ER−PR−/HER2+ and ER−PR−/HER2− (i.e. triple-negative breast cancers, TNBC).

Tumour response was assessed in the excision specimens by expert pathologists. Three categories were defined: (i) pathological complete response (pCR) with or without the presence of ductal carcinoma in situ (DCIS); (ii) partial response to therapy; ranging from minimal residual disease up to greater than 50 % of tumour cellularity; and (iii) no evidence of response. The last two categories were considered pathological non-complete response (pNCR).

### Statistical analysis

All statistics were calculated from the logarithm of the parameters, assessed for normality using the Shapiro–Wilk test and back-transformed where appropriate [24]. The mean value ± standard deviation of ADC and the non-Gaussian parameters were reported.

The pretreatment characteristics between pCR and pNCR were compared using the unpaired two-tailed *t* test.

The IHC subtypes were compared between response groups at baseline using the Chi-squared test. The imaging parameters across the four subtypes were also compared using the one-way analysis of variance (ANOVA). The correlation between the diffusion coefficients was evaluated using Pearson’s correlation. Receiver operating characteristics (ROC) curves were constructed to assess the performance of parameters in differentiating between pCR and pNCR, and areas under the curve (AUCs) were compared.

Repeatability of the baseline measurements was assessed at inter- and intraobserver levels using the intraclass correlation coefficient (ICC).

Model comparisons were made using the corrected Akaike information criterion (AICc), which imposes a penalty for additional parameters in the model [25]. The AICc was computed for the three models and averaged over all lesion voxels, which was subsequently averaged over all subjects. Statistical analyses were performed using the software SPSS (v. 21.0, Chicago, IL). As this study is primarily descriptive, *p* values are presented as raw values and not corrected for multiple comparisons. For purposes of discussion and similar to Orton et al. [5], multiple comparisons were accounted for by adjusting the *p* value significance threshold by a correction factor of 5. Thus statistically significant comparisons were set at *p* < 0.05 (no correction) and highly significant comparisons at *p* < 0.01.

## Results

### Baseline assessment

Four patients opted out of the study prior to imaging, therefore baseline analysis included 36 patients with unifocal malignant lesions. Twenty-five lesions (69 %) were classified as mass enhancement while 11 were non-mass-like lesions (31 %). All lesions showed heterogeneous enhancement on DCE-MRI; 27 lesions (75 %) demonstrated a predominately type III enhancement curve while the rest (9/36, 25 %) showed type II pattern. After surgery, 14 patients (39 %) showed pCR and 22 (61 %) were pNCR.

#### Prediction of response: pretreatment tumour characteristics

The mean tumour diameter was 4.7 cm (median 4.0 cm, range 1.2 − 12 cm). No significant difference was found between responders (4.1 ± 0.4 cm) and non-responders (5.1 ± 0.5 cm, *p* = 0.194).

According to IHC subtype, 33 % (12/36) were ER+PR+/HER−, 33 % ER+PR+/HER2+, 3 % (1/36) ER−PR−/HER2+, and 31 % (11/36) were categorised as TNBCs.

All but one patient in the triple-negative group showed pCR, whereas 17 % (2/12) of both ER+PR+/HER2− and ER+PR+/HER2+ tumours showed complete response.

Significant differences were observed between response groups with respect to histological subtype (*p* < 0.001).

#### Prediction of response: pretreatment diffusion parameters

**Fig. 1:**
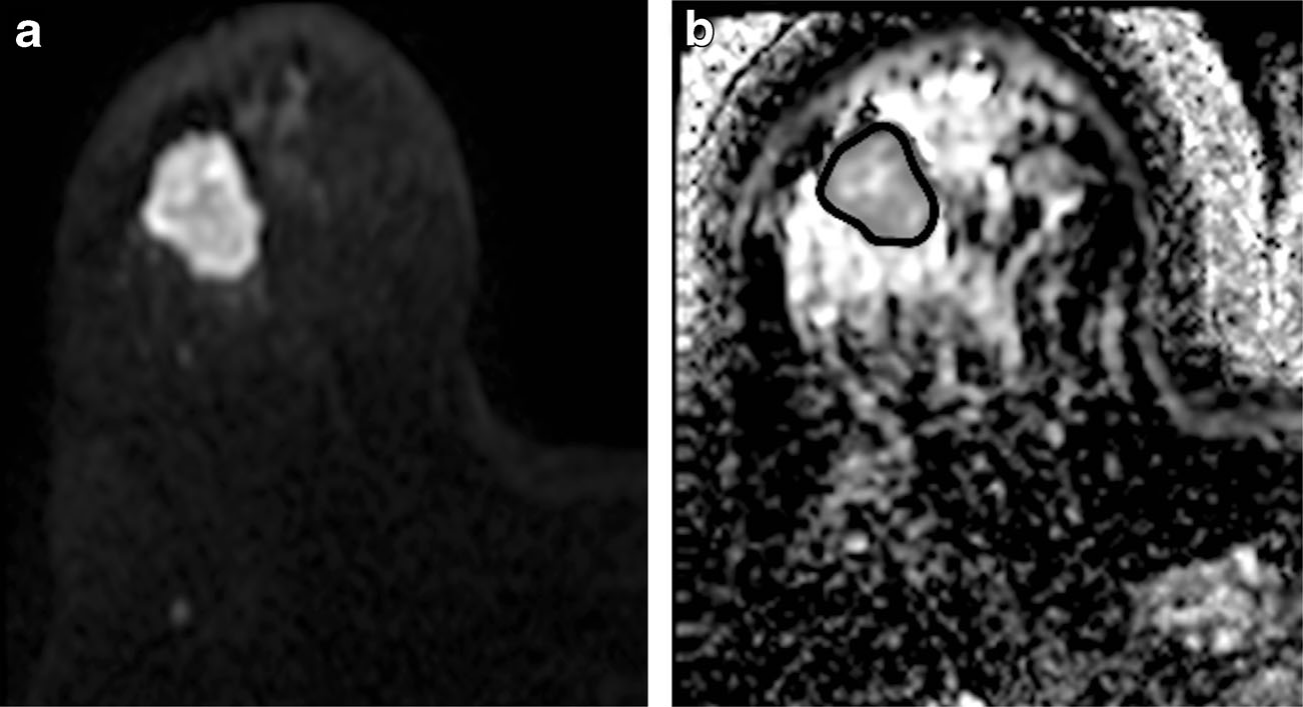
Representative images from pretreatment MRI of a 49-year-old female patient with cancer of the right breast: **a** axial DW image showing hyperintense tumour with restricted diffusion on the *b*900 s/mm^2^ image. **b** ADC map was generated from two *b* values (0, 900 s/mm^2^). ROI was drawn on the primary lesion and copied to the ADC map (ADC = 0.92 ± 0.094 × 10^−3^ mm^2^/s)

**Fig. 2:**
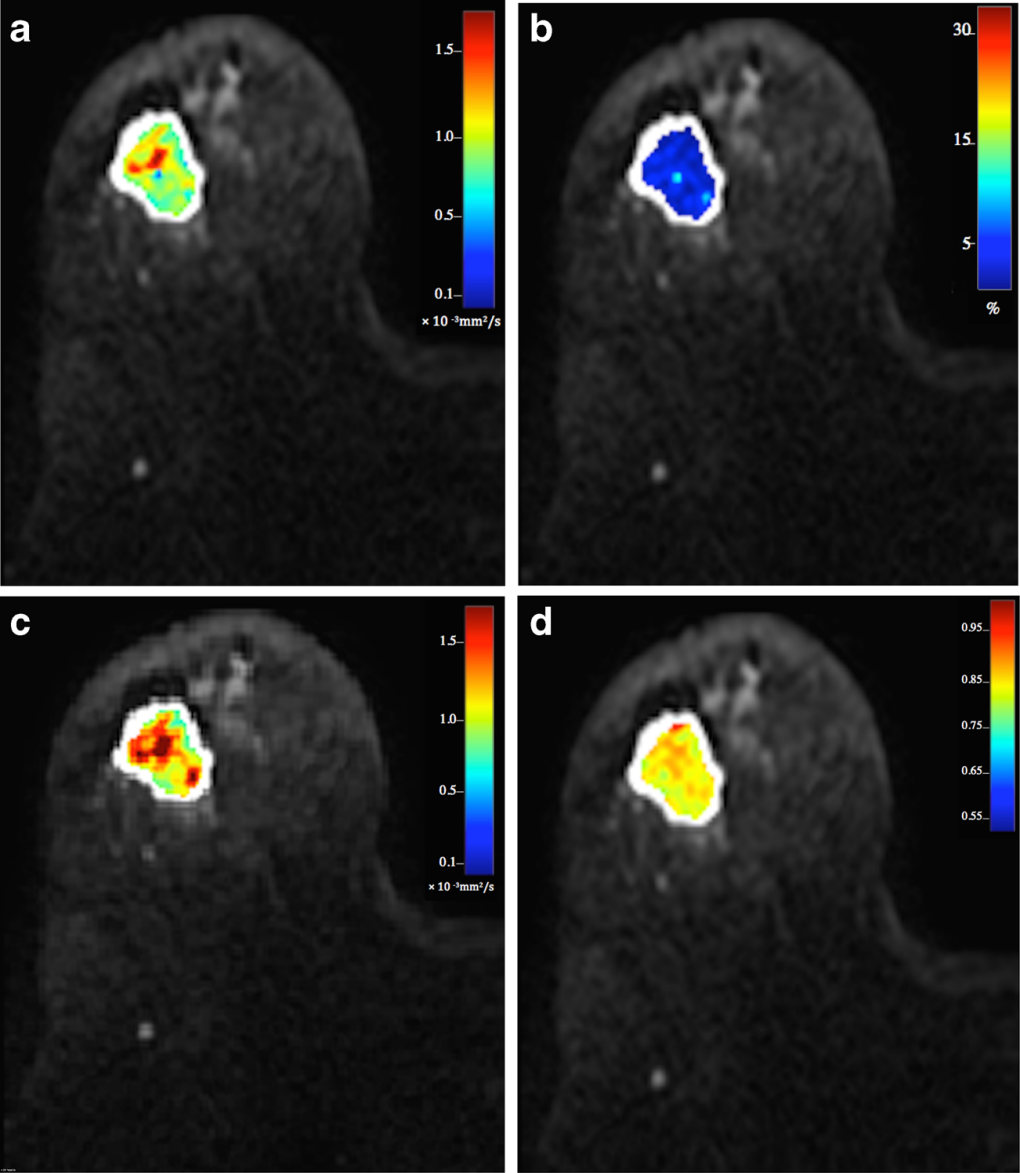
Parametric maps of the bi-exponential and stretched-exponential models in the same female patient as in Fig. 1: **a** *D*_t_ = 0.80 ± 0.28 × 10^−3^ mm^2^/s, **b** ƒ = 11.8 ± 1.3 %, **c** DDC = 0.98 ± 0.12 × 10^−3^ mm^2^/s, **d** α = 0.84 ± 0.18. It should be noted that the α values in tumours express the intravoxel heterogeneity, whereas the other maps show intervoxel heterogeneity between tissues. At histopathology, the lesion was identified as a grade 3 invasive ductal carcinoma of TNBC subtype. The patient underwent wide local excision of the lesion. On the excision specimen, no invasive components were seen and the patient was considered a complete pathological responder to NACT

**Fig. 3:**
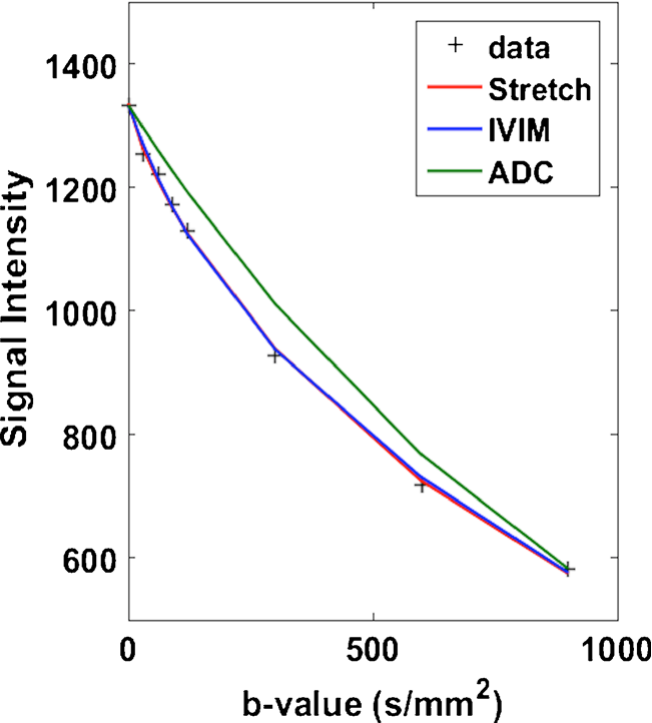
Mono-exponential (*green*), bi-exponential (*blue*) and stretched-exponential curves (*red*) fitted to one pixel in a breast lesion in a pretreatment scan

Pretreatment mean ADC, DDC and *D*_t_ values across the cohort were 1.11 × 10^−3^, 1.09 × 10^−3^ and 0.93 × 10^−3^ mm^2^/s respectively. Figures 1 and 2 show representative images and parametric maps of a complete responder. An example of the mono-, bi- and stretched-exponential curves fitted to the data from one pixel is illustrated in Fig. 3.

Table 3 shows the mean diffusion coefficients of the response groups from all three models. Responders showed significantly lower ADC, DDC and *D*_t_ values (0.92 ± 0.03, 0.93 ± 0.04 and 0.85 ± 0.05 × 10^−3^mm^2^/s respectively) relative to non-responders (1.20 ± 0.02, 1.25 ± 0.03 and 1.02 ± 0.05 × 10^−3^mm^2^/s respectively) (*p* < 0.01, *p* < 0.01, *p* = 0.02 respectively). The ƒ fraction showed higher fractions in responders; however, this was not significant (*p* = 0.09). On subgroup analysis, ƒ was found to be significantly higher in responders of the TNBC subtype (12.4 ± 4.1 % vs. 10.9 ± 1.2 %, *p* = 0.01).

**Table 3:** Mean diffusion parameters according to patient outcome

Parameters	Responders	Non-responders	*p* value
Baseline (*n* = 36)			
ADC (×10^−3^mm^2^/s)	0.92 ± 0.03	1.20 ± 0.02	<0.01**
DDC (×10^−3^mm^2^/s)	0.93 ± 0.04	1.25 ± 0.03	<0.01**
α	0.84 ± 0.02	0.81 ± 0.02	0.07
*D*_t_ (×10^−3^mm^2^/s)	0.85 ± 0.05	1.02 ± 0.05	0.02*
ƒ (%)	12.10 ± 2.02	10.32 ± 1.15	0.09
Mid-treatment (*n* = 22)			
Parameters	Responders (% change)	Non-responders (% change)	*p* value of %change
ADC (×10^−3^mm^2^/s)	1.52 ± 0.32 (↑49 %)	1.27 ± 0.18 (↑21 %)	0.03*
DDC (×10^−3^mm^2^/s)	1.51 ± 0.15 (↑43 %)	1.40 ± 0.12 (↑32 %)	0.04*
α	0.91 ± 0.07 (↑7 %)	0.86 ± 0.11 (↑5 %)	0.68
*D*_t_ (×10^−3^mm^2^/s)	1.30 ± 0.14 (↑36 %)	1.28 ± 0.15 (↑23 %)	0.14
ƒ (%)	8.48 ± 1.54 (↓29 %)	10.53 ± 2.51 (↑5 %)	0.05

Baseline and mid-treatment values of the various parameters (units × 10^−3^ mm^2^/s except where shown). Values in parenthesis are the percentage change. *P* values using a two-tailed independent *t* test compared between response groups. Significant statistics have *p* < 0.05, while highly significant statistics have *p* < 0.01 which includes a correction for multiple comparisons). Unless otherwise indicated, data represent mean values ± standard deviation (percentage change between pretreatment and mid-treatment values) of the mono-exponential (ADC), bi-exponential (*D*_t_, ƒ) and stretched-exponential (DDC, α) parameters

*Values show statistically significant differences

**Values show highly significant differences

Although lower α values were found in non-responders compared to eventual responders, this was not significant (0.81 ± 0.02 vs. 0.84 ± 0.02, *p* = 0.07).

**Fig. 4:**
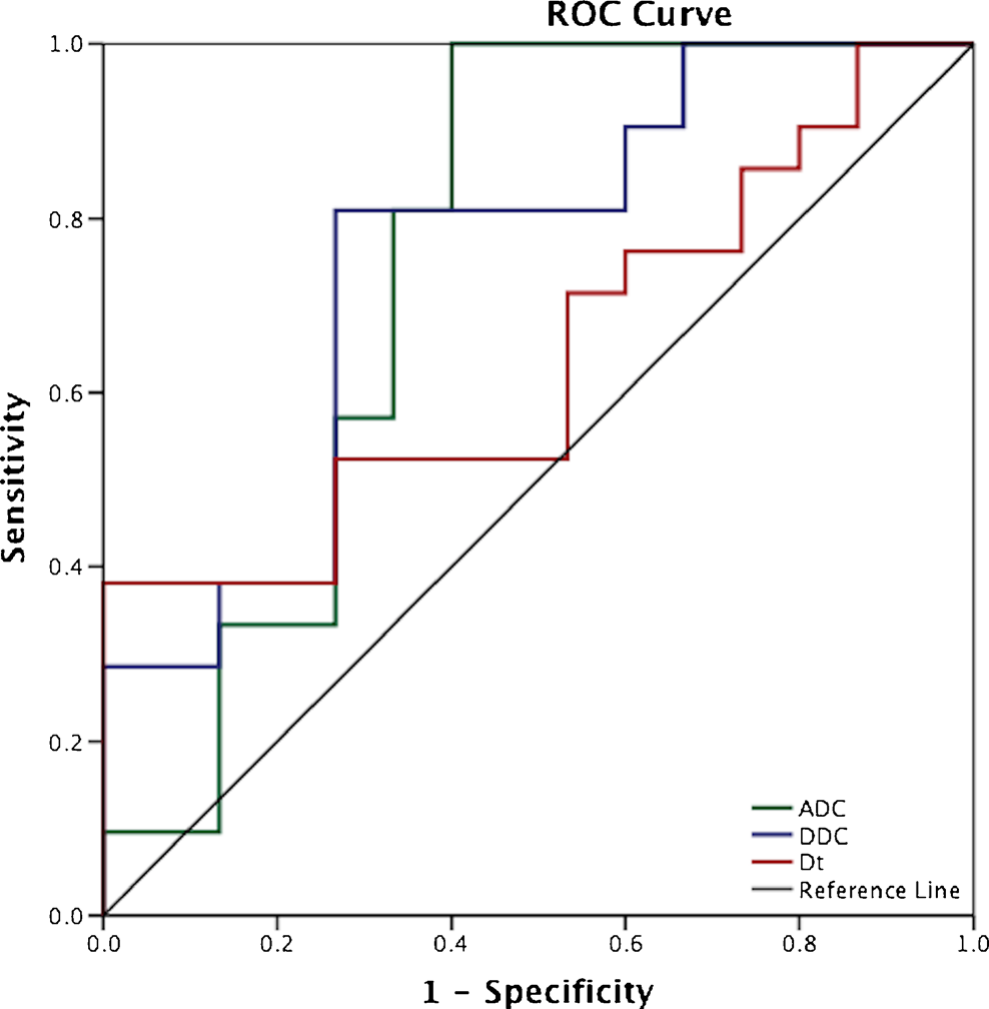
ROCs for the response prediction of the pretreatment diffusion coefficients from the mono-exponential, bi-exponential and stretched-exponential models. The DDC demonstrated the largest AUC (0.75, *p* = 0.01) compared with ADC and *D*_t_ (0.74, *p* = 0.01 and 0.641, *p* = 0.15 respectively). The cut-off to differentiate between response groups on pretreatment MRI for DDC (1.141 × 10^−3^ mm^2^/s) yielded the highest measures of accuracy (sensitivity 81 %, specificity 72 %)

Figure 4 shows the ROC curves comparing the diagnostic efficacy of diffusion coefficients relative to pathological response. DDC demonstrated a larger AUC (0.756, *p* = 0.01) compared to ADC and *D*_t_ (0.749, *p* = 0.01 and 0.641, *p* = 0.15 respectively). The DDC cut-off to differentiate response groups on pretreatment MRI (1.141 × 10^−3^ mm^2^/s) yielded the highest sensitivity (81 %) and specificity (72 %) (Table 4).

**Table 4:** ROC analysis of the various parameters (units × 10^−3^ mm^2^/s except where shown) indicating the sensitivity and specificity measures at the respective cut-off values and their significance levels

Parameter	AUC	Cut-off value (×10^−3^mm^2^/s)	Sensitivity (%)	Specificity (%)	*p* value
ADC (×10^−3^mm^2^/s)	0.749	1.012	81	67	0.01*
DDC (×10^−3^mm^2^/s)	0.756	1.141	81	72	0.01*
*D*_t_ (×10^−3^mm^2^/s)	0.641	0.967	71	53	0.15
α (unit-less)	0.644	0.838	60	47	0.14
ƒ (%)	0.637	11.01	66	43	0.16

Values show statistical significance of the pretreatment mono-exponential (ADC), bi-exponential (*D*_t_, ƒ) and stretched-exponential (DDC, α) parameters

*AUC* area under the curve

### Mid-treatment assessment

All patients attended a second MRI midway through treatment; however, 14 patients were excluded for the following reasons: patients opted out of the research examination (*n* = 3), images were unusable as a result of suboptimal fat suppression (*n* = 2), subjects not receiving the standard six cycles of chemotherapy (*n* = 2), three patients did not proceed with the full diffusion imaging sequence prior to surgery after NACT as they found it intolerable. Additionally, four patients were excluded from analysis as their follow-up MRI was not performed in a timely manner at mid-therapy (post cycle 3). These patients developed liver and kidney lesions that were later found to be benign. Finally, 22 malignant lesions were available for analysis on the midway examination. Eight patients (36 %) showed pCR and 14 (64 %) were pNCRs.

#### Prediction of response: change in tumour size

A significant difference was found in tumour size as responders showed a smaller mean tumour size (1.5 ± 0.2 cm vs. 2.9 ± 0.5 cm, *p* < 0.05).

#### Prediction of response: change in diffusion parameters

**Fig. 5:**
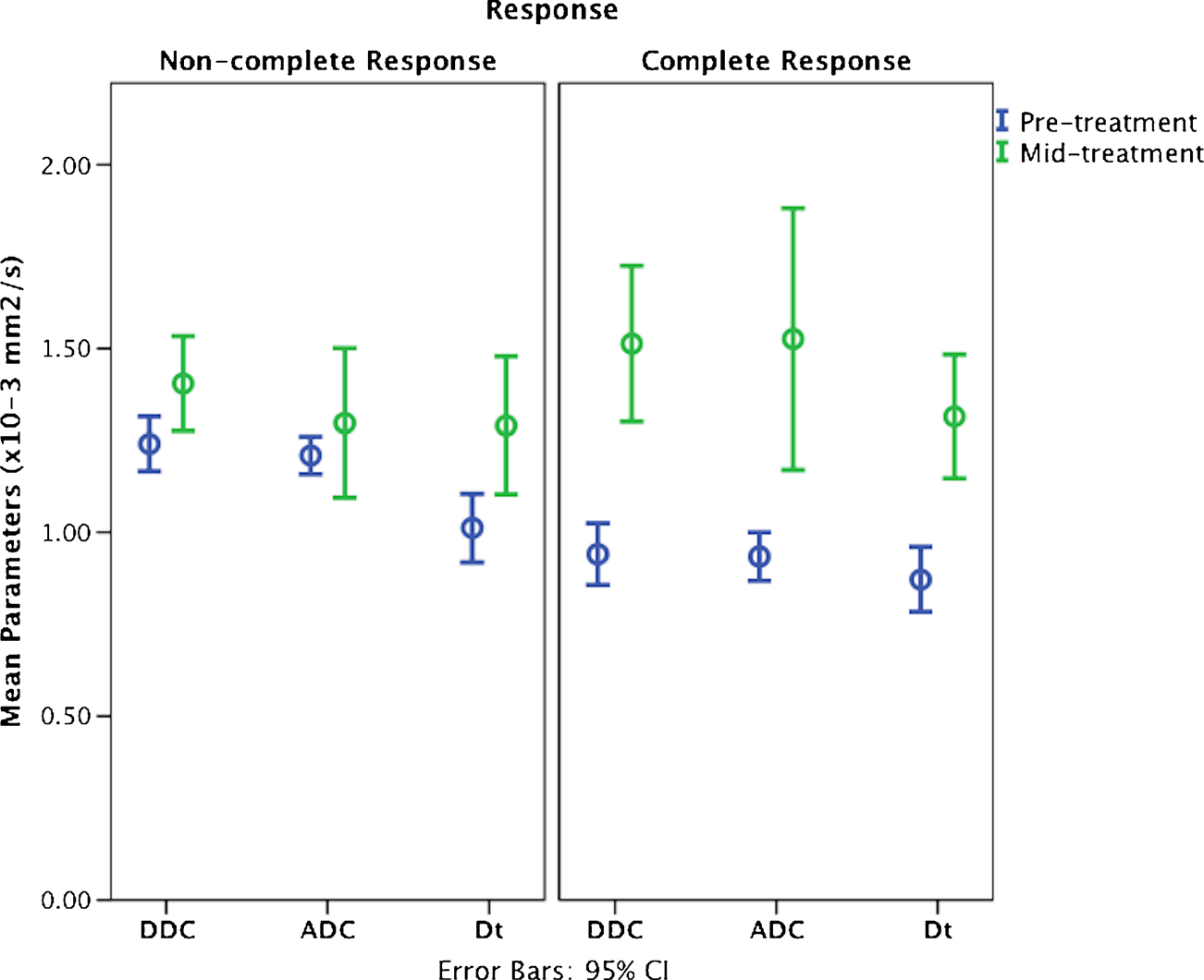
Mean distribution of the diffusion coefficients of the three models before the start of treatment and after three cycles of chemotherapy in complete and non-complete responders. There is an overall increase in parameters in both groups; however, a larger increase is noted in the ADC and DDC of complete responders. When the increase in mean values of ADC and DDC were compared between response groups at mid-treatment, a significant difference was observed; *p* = 0.03 and *p* = 0.04 respectively. However, the increase in *D*_t_ did not show statistical significance between response groups (*p* = 0.14). *Error bars* represent the 95 % confidence interval

An increase in the mean values of the diffusion coefficients was observed after three cycles of chemotherapy, with the percentage increase in ADC and DDC showing a statistically significant difference between responders (49 % and 43 %) and non-responders (21 %, 32 %, *p* = 0.03 and *p* = 0.04 respectively). However the increase in *D*_t_ did not show a significant difference between response groups (36 % vs. 23 %, *p* = 0.14). Moreover, the decrease in ƒ fraction found in responders (29 %) was substantially different from the *increase* in ƒ observed in pNCR (5 %, *p* = 0.05). Responders also showed a larger increase in α compared to pNCR (7 % vs. 5 %). This, however, was not significant (*p* = 0.68) (Table 3). Figure 5 shows the mean change in the diffusion coefficients between response groups.

### Repeatability of measurements

Overall parameters of the mono- and stretched-exponential models showed excellent inter- and intrarater repeatability. Bi-exponential parameters ranged from excellent to fair (Table 5).

**Table 5:** Inter- and intraobserver repeatability measures of the diffusion parameters using the intraclass correlation (ICC) metrics

Parameter	Interobserver agreement	Intraobserver agreement
ADC (×10^−3^mm^2^/s)	0.816 (0.657, 0.912)	0.910 (0.823, 0.954)
DDC (×10^−3^mm^2^/s)	0.789 (0.601, 0.898)	0.860 (0.725, 0.929)
*D*_t_ (×10^−3^mm^2^/s)	0.699 (0.641, 0.859)	0.778 (0.756, 0.889)
α (unit-less)	0.808 (0.760, 0.938)	0.822 (0.648, 0.910)
ƒ (%)	0.605 (0.569, 0.868)	0.695 (0.531, 0.893)

Data represents intraclass correlation with 95 % confidence intervals

### Model selection

A significant correlation was found between ADC and the non-Gaussian diffusion coefficients (*p* < 0.001 for both). A higher correlation, however, was observed between ADC and DDC (*r* = 0.89) compared to ADC and *D*_t_ (*r* = 0.76).

The AICc from the ROI data showed that stretched-exponential was the preferred model at baseline and mid-treatment measurements, showing lower values (62.6 ± 8.2, 66.6 ± 9.2) compared to the mono-exponential and bi-exponential models (baseline, 81.7 ± 9.4 and 82.9 ± 9.7; mid-treatment, 85.6 ± 10.2 and 88.6 ± 11.6 respectively).

## Discussion

It is generally accepted that highly proliferating malignant breast lesions result in a packed cellular microstructure, showing more restricted diffusion and decreased ADC [26]. However the choice of *b* value may affect the calculated measurement, which is influenced by multiple pools diffusing at different rates, confounding the assessment of tissue diffusivity [27]. Alternative diffusion models have been devised to account for the more complex non-Gaussian diffusion behaviour of biological tissues in vivo. Le Bihan et al. have shown that signal attenuation is not only a result of random microscopic motion of water molecules influenced by cell density but is also dependent on microperfusion within the voxel [6]. This has become of particular interest, as tumour angiogenesis is seen an important determinant in the outcome of patients. Numerically, however, it has been shown that fitting three parameters for the bi-exponential model can be unstable [28, 29]. In this study we assessed two non-Gaussian models acquired at eight *b* values to capture the various diffusion properties of tissues and compared them with the mono-exponential model using the standard *b* values commonly implemented in DW-MRI of the breast.

Our baseline results were consistent with Sigmund et al. showing lower *D*_t_ values compared to ADC [30]. This was expected on exclusion of the perfusion effect, as the lower *b* values were employed to capture the much higher pseudo-diffusion. Furthermore, a strong correlation was found between the mono- and stretched-exponential diffusion coefficients. This suggests that DDC can be interpreted in the same way as ADC with the observation of a continuous distribution of diffusion components within the microenvironment.

Similar to Park et al. [31], our study also shows an inverse correlation between tumour diffusion coefficients and therapeutic response, where substantially higher pretreatment values of ADC, DDC, *D*_t_ and lower ƒ fractions were more suggestive of necrotic, less viable lesions. These lesions have often been found to be hypoxic, more aggressive and less sensitive to chemotherapy [32].

The mean ƒ fraction of tumours was also able to differentiate responders in the most biologically aggressive subtype (TNBC), showing complete response in about one-third of tumours, consistent with previous reports [33]. However, a full statistical evaluation was not possible because of the small sample size of the other subtypes.

Although the attribution of the diffusion index α to a biological correlate is still under investigation, lower values seen in non-responsive lesions indicate a more heterogeneous microenvironment *within* the imaging voxels [19]. This parameter could in turn be viewed as a reflection of the microstructural complexity of the tissue, suggesting changes in the degree of cellular pleomorphism, vascular heterogeneity and presence of microscopic necrosis [13].

Although non-significant (*p* = 0.68), responders in our study showed an increase in the structural homogeneity (α closer to 1) at mid-treatment compared to non-responders. These preliminary results are similar to those found by Orton et al. in abdominal and pelvic tumours where changes in α were substantially different between response groups when assessed before the start of therapy, 7 days and 28 following treatment [5].

Neoadjuvant chemotherapy regimens used in the treatment of breast cancer commonly consist of an anthracycline in combination or sequentially administered with taxanes. The mechanism of action of anthracyclines is mainly attributed to the inhibition of DNA synthesis, preventing the replication of rapidly dividing cells [34]^,^ whereas taxanes have been reported to have an anti-angiogenic effect with selective shutdown of microvessel formation [35, 36]. This is supported by the significant increase in DDC seen on mid-treatment (*p* = 0.04), suggesting a change in the distribution of diffusion compartments resulting from the early breakdown of the vascular endothelial cells. On IVIM analysis, we also showed a large decrease in the perfusion fraction in lesions showing complete response, which is in contrast to its increase in lesions failing to respond to treatment. This result is supported by previous findings [37, 38].

When repeatability of measurements was assessed, the mono- and stretched-exponential showed high ICC measures, suggesting equivalent robustness of the derived parameters to ADC. This is consistent with recent studies that have found the stretched exponential equivalent to or outperforming other models [5, 12, 16, 39].

This work suffered a few limitations. First, the chemotherapy regimen in our population varied, which made it difficult to draw specific conclusions on the biological effect of each drug as detected by the diffusion models. However, this should not have impacted the final response assessment as the population received the same combination by the end of NACT.

Second, although we evaluated the repeatability of the ROI measurements, we did not perform validation studies. Orton et al., however, showed that the parameters derived from the stretched-exponential model are highly reproducible and could potentially serve as quantitative biomarkers for response assessment in abdominal and pelvic tumours [5].

In this study, we implemented the stretched-exponential model using eight *b* values to characterise the deviation from the Gaussian curve and compared it with the bi-exponential model. Past studies have used 4−5 *b* values and produced similar results [13, 14, 19], which affords the potential of increased DW-MRI capability for characterising tissue properties over an extended *b* value range at reasonable scan times.

Finally, our study was limited by the small sample size with differing rates of responders within the tumour subtypes. However, we showed that the DW-MRI could be sensitive to baseline and early treatment effects caused by NACT using the DDC parameter of the stretched-exponential model, as demonstrated by the large AUC with satisfactory sensitivity and specificity measures, and the ƒ fraction of the bi-exponential model. Unlike in abdominal and pelvic tumours [5], α did not differentiate between response groups in the breast. This may relate to the timing of the follow-up MRI, which was performed after the third cycle of NACT (i.e. 6 weeks of treatment). Therefore follow-up imaging at an earlier time point may elucidate the change in α between responders and non-responders.

In conclusion, this feasibility study showed that DW-MRI is sensitive to baseline and early treatment changes in breast cancer using non-mono-exponential models, which offer additional imaging biomarkers that can potentially provide insights into the cellular compartments and membranes and may become more sensitive to treatment-induced tissue changes. Our results show that the stretched-exponential model can potentially monitor such changes. This data supports the wider use of these models in assessing treatment effects beyond that routinely measured with ADC.

## Electronic supplementary material

Below is the link to the electronic supplementary material.

ESM 1 (DOCX 81 kb)

## Abbreviations

α: Alpha, Intravoxel heterogeneity index
ADC: Apparent diffusion coefficient
AICc: Corrected Akaike information criterion
ANOVA: One-way analysis of variance
AUCs: Areas under the curves
DCE: Dynamic contrast-enhanced
DDC: Distributed diffusion coefficient
*D*_p_: Pseudo-diffusion coefficient
*D*_t_: Tissue diffusion coefficient
DW-MRI: Diffusion-weighted magnetic resonance imaging
ER: Oestrogen receptor
ƒ: Vascular fraction
HER2: Human epidermal growth factor 2
ICC: Intraclass correlation coefficient
IHC: Immunohistochemical
IVIM: Intravoxel incoherent motion
NACT: Neoadjuvant chemotherapy
pCR: Complete pathological response
pNCR: Pathological non-complete response
PR: Progesterone receptor
ROC: Receiver operating characteristics curves
TNBC: Triple-negative breast cancers
