# Supplementary material for: Assessment of early treatment response to neoadjuvant chemotherapy in breast cancer using non-mono-exponential diffusion models: a feasibility study comparing the baseline and mid-treatment MRI examinations
Source: Eur Radiol. 2016 Oct 31;27(7):2726–36. doi: 10.1007/s00330-016-4630-x (PMC5486805; doi:10.1007/s00330-016-4630-x)
Supplement: Supplementary file 1 — (DOCX 81 kb) [file 330_2016_4630_MOESM1_ESM.docx]

**Supplementary material:**

The classical mono-exponential and the non-Gaussian bi-exponential and stretched exponential models are expressed mathematically as follows:

1. Classical mono-exponential diffusion model:

$S\left( b \right)=S\left( 0 \right).\exp(-b .ADC)$ (1)

where S(b) and S(0) denote the signal intensity obtained with the diffusion gradient b-value of b and in absence of a diffusion gradient respectively. The apparent diffusion coefficient (ADC) is the sole parameter to be estimated through fitting to this model.

1. Bi-exponential IVIM model:

$S\left( b \right)= S\left( 0 \right). [\left( 1-ƒ \right)\exp\left( -b . Dt \right)+ ƒ \exp\left( -b . Dp \right)]$ (2)

where Dt is the true diffusion coefficient, ƒ is the perfusion volume fraction and Dp is the pseudo-diffusion coefficient influenced by the capillary geometry and blood velocity. The DWI data fitting was performed using the segmented approach, which provides more accurate and robust estimation than the full fitting of DWI signals to the bi-exponential function. Dt was first obtained with a least-square fitting to a mono-exponential function by using the data points at b-values over 200 s/mm^2^. The fitted curve was then extrapolated to obtain an intercept at b=0. Perfusion fraction, ƒ, was estimated from ratio between this intercept and the signal intensity at b=0. Finally, Dp was calculated from a bi-exponential fit with constrained Dt and fp according to Eq. 2.

1. Stretched exponential model:

$S\left( b \right)=S\left( 0 \right){\exp(-b . DDC)}^{\alpha}$ (3)

where DDC is the distributed diffusion coefficient and α is the unit-less stretching parameter or heterogeneity index (0 ≤ α ≤ 1). In the case of homogenous diffusion α =1, and the function indicates a mono-exponential decay of the diffusion signal described by the first model. Lower values of α result from non-mono-exponential behaviour caused by multiple proton pools with a range of diffusion rates within the imaged voxel.
